# Oil Content and Fatty Acid Composition of Safflower (*Carthamus tinctorius* L.) Germplasm

**DOI:** 10.3390/foods14020264

**Published:** 2025-01-15

**Authors:** Cemal Kurt, Muhammad Tanveer Altaf, Waqas Liaqat, Muhammad Azhar Nadeem, Ayşe Nuran Çil, Faheem Shehzad Baloch

**Affiliations:** 1 Department of Field Crops, Faculty of Agriculture, Çukurova University, Adana 01330, Türkiye; ckurt@cu.edu.tr (C.K.); waqasliaqat0043@gmail.com (W.L.); 2 Department of Field Crops, Faculty of Agriculture, Recep Tayyip Erdoğan University, Rize 53300, Türkiye; 3 Department of Plant Production and Technologies, Faculty of Agricultural Sciences and Technologies, Sivas University of Science and Technology, Sivas 58140, Türkiye; azharjoiya22@gmail.com; 4 Eastern Mediterranean Agricultural Research Institute, Adana 01321, Türkiye; aysenuran.cil@tarimorman.gov.tr; 5 Department of Biotechnology, Faculty of Science, Mersin University, Yenişehir, Mersin 33343, Türkiye; 6 Department of Plant Resources and Environment, Jeju National University, Jeju 63243, Republic of Korea

**Keywords:** safflower, fatty acid, oil content, linoleic acid, germplasm diversity, breeding programs, sustainable production

## Abstract

Safflower (*Carthamus tinctorius* L.) is a promising oilseed crop with potential applications in the food, pharmaceutical, and industrial sectors. Understanding the oil content and fatty acid composition of safflower germplasm is crucial for breeding programs aimed at enhancing its agronomic and nutritional traits. This study assessed the oil content and fatty acid composition in 87 safflower accessions. Significant variations were observed, with the oil content ranging from 36.88% to 18.44%. Genotype Egypt 1 exhibited the highest oil content. Among fatty acids, China 1 had the highest myristic acid (0.170%) content, while Remzibey had the lowest (0.100%). Palmitic acid ranged from 6.13% to 8.20%, with Egypt 3 and Bangladesh 3 at the extremes. For palmitoleic acid, Jordan 5 had the highest content (0.53%) and Bangladesh 2/Portugal 2 the lowest (0.03%). Linoleic acid varied from 37.7% (China 7) to 77.73% (Iran 1). A correlation analysis indicated strong positive correlations between protein and oil content, as well as between palmitic and myristic acids, and between palmitic and linoleic acids. Conversely, protein exhibited highly negative correlations with myristic, palmitic, and palmitoleic acids. The protein percentage showed a high heritability but a low genetic advance, while palmitic acid, oil percentage, stearic acid, linoleic acid, palmitoleic acid, and oleic acid showed a high heritability and a moderate genetic advance as a percentage of the mean. These findings can aid in developing cultivars with enhanced fatty acids, oil quality, and nutritional value, facilitating sustainable production for a wide range of industrial applications.

## 1. Introduction

Safflower (*Carthamus tinctorius* L.) is a plant belonging to the Compositeae family with a chromosome number of 2n = 24. It has a long history of cultivation as an oil crop in the Middle East, dating back approximately 3000 years [1]. Safflower is primarily cultivated in arid agricultural regions to produce cooking oil, as it exhibits a greater adaptability to dry conditions and lower rainfall compared to other oil crops. Additionally, safflower demonstrates relatively favorable resistance to cold temperatures [2]. This minor oilseed crop exhibits versatility and holds the potential to provide numerous advantages to rainfed cereal-based cropping systems. This is primarily attributed to its ability to withstand adverse environmental conditions such as cold, drought, and salinity, as well as its reduced dependency on external inputs [3,4,5]. The cultivation of this plant is feasible in arid, semi-arid, and rainfed environments due to its extensive root system [6]. Safflower exhibits the capacity to thrive under water stress without experiencing a substantial decline in oil and seed yield, thus reinforcing its status as a promising crop in such challenging environments.

Comparatively, safflower exhibits enhanced disease resistance and the ability to establish soil cover earlier, thereby reducing the risks associated with nitrogen leaching and soil erosion, when compared to sunflower. Recently, safflower has garnered attention from researchers due to the intriguing characteristics of its oil for both food and non-food applications [7,8]. Safflower is grown in over 25 countries. However, the Russian Federation, Kazakhstan, India, Argentina, Mexico, the USA, Uzbekistan, and Türkiye are the primary countries cultivating safflower [9]. The cultivation of safflower exhibits significant growth potential in Turkey, particularly in regions characterized by continental and Mediterranean climates [10,11]. The provision of substantial agricultural subsidies by the Turkish government to safflower producers, with the aim of decreasing Turkey’s reliance on imported vegetable oil has played a crucial role in the advancement of safflower cultivation in Türkiye [10]. The plant possesses significant economic and medicinal value due to its seed oil and extract from its flowers [12].

The efficacy of safflower oil in various applications such as food, pharmaceutical, and cosmetic industries has been substantiated by multiple studies [13,14,15,16]. The significance of this crop is primarily supported by its high content of unsaturated fatty acids, specifically oleic and linoleic acids [17,18,19,20]. The consumption of safflower seed oil can provide various health advantages to individuals due to its elevated concentration of unsaturated fatty acids. As an illustration, the functional activities associated with monounsaturated fatty acid, specifically oleic acid, encompass a reduction in cardiovascular disease risk through the lowering of systolic blood pressure. This acid is preferred by consumers due to its notable stability and bland flavor [21].

The introduction of this crop to semi-arid regions could serve as an alternative for the development of oilseed crops due to its strong ability to adapt to drought and high temperatures [22]. The efficacy of this approach necessitates research to assess the impact of this context on the quality of the supplied oil. The composition of safflower oil primarily consists of unsaturated fatty acids, namely linoleic (C18:2), oleic (C18:1), and linolenic acids (C18:3), with a relatively low proportion of saturated fatty acids. Among the saturated fatty acids present, palmitic (C16:0) and stearic (C18:0) are the most abundant [16]. Safflower oil is known for its high tocopherol content, which contributes to its functional properties as a food with potential anti-cholesterol effects and cardiovascular protective benefits [23,24].

Safflower oil has been identified as a potential source of natural polymers that can be utilized in various industrial and pharmaceutical applications [24,25]. In addition, the substantial quantities of safflower by-products, such as seed meal, have the potential to be utilized as feed rations for livestock [26] and energy production [27], thereby augmenting the overall value chain of the safflower crop. The yield and fatty acid composition of safflower, which determine the crop’s suitability for nutritional, industrial, or pharmaceutical applications, are significantly influenced by the genotype [28] as well as agronomic practices including irrigation, fertilization, sowing date, and harvest date [29,30,31,32].

Similarly, the combination of genotypes and environmental factors, specifically moisture and temperature, during the process of seed maturation has an impact on the synthesis of fatty acids and the relative proportions of oleic and linoleic acids in the seeds [33,34]. Safflower has demonstrated significant adaptability to the arid and semi-arid regions of the Mediterranean region, making it a potential candidate for inclusion in crop rotation systems alongside winter wheat or annual legumes [35,36]. The incorporation of safflower as an alternative and drought-tolerant oilseed crop into conventional rainfed cereal-based cropping systems presents a viable option that can potentially enhance the productivity of agricultural systems and enable farmers to mitigate the adverse effects of climate variability and economic uncertainties. The current study involved a comparison of 87 genotypes to assess their seed quality characteristics, specifically focusing on the composition of oil, protein, and fatty acids. The acquired information will prove valuable to safflower breeders in devising effective breeding strategies for the development of novel and improved cultivars.

## 2. Materials and Methods

Eighty-seven distinct safflower genotypes, sourced from various countries, constituted the plant material for this investigation (Table 1). The accessions used in the present research were provided by Plant Genetic Resources Institute (PGRI) Pakistan, The Turkish Central Research Institute for Field Crops, and the United States Department of Agriculture (USDA) [37].

### 2.1. Experimental Design and Statistical Analysis

**Figure 1:**
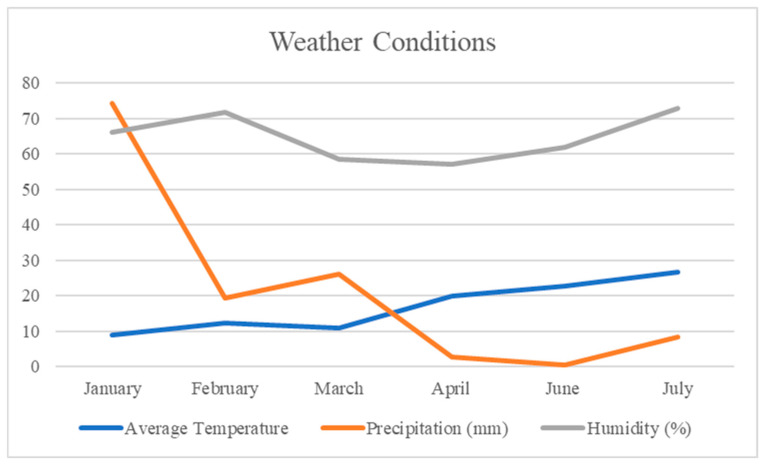
Weather conditions during the experiment.

The field experiment was conducted according to an augmented block design at the experimental area of Çukurova University in Adana, Türkiye, which has a typical Mediterranean climate characterized by mild, rainy winters and hot, dry summers [38]. This was a one-year field study performed under the natural prevailing conditions of the region, which were consistent for all genotypes under study (Figure 1). To ensure uniform conditions across all genotypes, the experiment was conducted within a single growing season, with all genotypes planted in the field simultaneously. Standard agronomic practices were applied consistently throughout the study.

Fertilizers were applied uniformly before planting at a rate of 250 kg/ha of 20-20-0 (providing 50 kg/ha N and 50 kg/ha P) alongside urea (46% N) at 200 kg/ha. Sowing was carried out manually in the second week of December 2021, with each accession allotted a 5 m row length, a row spacing of 70 cm, and an intra-row spacing of 15 cm. Harvesting was conducted in the last week of June 2022. Although environmental factors were not artificially controlled, conditions such as temperature and rainfall were monitored to ensure consistency, thus minimizing any environmental variation that could influence the results.

### 2.2. Oil Extraction and GC Analysis

The samples of 87 safflower genotypes were subjected to oil extraction using a Soxhlet apparatus and the gravimetric method. For oil content determination, 5 g of clean, mature seed samples was weighed and extracted in triplicate to assess repeatability. The Soxhlet apparatus (FOSS) was used with petroleum ether (99.9%, Merck, Rahway, NJ, USA) as the solvent, and extraction continued for 3 h following the method developed by Kurt [39]. The precision of the extraction was confirmed by calculating the coefficient of variation across triplicate samples, with values below 5% indicating a high repeatability of the method. This validation step aligns with Soxhlet extraction standards for oilseed analyses, confirming the reliability of the data produced.

An oil sample of 500 mg was dissolved in 2 mL isooctane followed by 1.5 mL of 0.5 M methanolic NaOH (99.6%, Sigma, Setagaya City, Japan). The tube was then vortexed, held in boiling water for 7 min, and cooled to room temperature. Two ml of BF3 (99.99% Boron trifluoride, Sigma) were added, vortexed, and held in boiling water for 5 min and allowed to come to room temperature. After adding 5 mL NaCl (Merck), the tube was vortexed, and centrifuged at 4000 rpm for 10 min. The supernatant was used for GC analyses [40].

The fatty acid (FA) composition was analyzed using a GC Clarus 500 with an auto sampler (Perkin Elmer, Waltham, MA, USA) equipped with a flame ionization detector and a fused silica capillary SGE column (30 m • 0.32 mm, ID • 0.25 lm, BP20 0.25 UM, USA). The oven temperature was brought to 140 °C for 5 min, then raised to 200 °C at a rate of 4 °C/min and to 220 °C at a rate of 1 °C/min, while the injector and the detector temperatures were set at 220 °C and 280 °C, respectively [39]. Moreover, FA analysis was conducted with rigorous quality control measures to ensure data consistency and accuracy. These included the use of internal standards and the routine calibration of the gas chromatography (GC) equipment, following established protocols [41,42]. Internal standards were added to each sample prior to extraction, and calibration of the GC was regularly performed to minimize analytical variation. These procedures ensured reliable comparisons across genotypes and accurately reflect the observed variation in the fatty acid composition.

### 2.3. Protein Analysis

Crude protein content was determined using the micro-Kjeldahl method with a FOSS autoanalyzer, including three independent replications to ensure accuracy. This method calculates protein content based on nitrogen quantification, with the results expressed as crude protein [43].

### 2.4. Statistical Analysis

The statistical analysis was performed according to an augmented randomized complete block design [44] using the statistical software R studio 4.3.2. The package used for analysis was the “Augmented RCBD” package [45], Moreover, broad-sense heritability and genetic advance as a percentage of the mean (GAM) were also estimated for all traits using R. The statistical software XLSTAT, version 2021.3.1 (www.xlstat.com) was used to perform descriptive analysis, including mean, maximum, minimum, and standard deviation calculations for all seven traits under investigation. Pearson’s correlation analysis was conducted to examine relationships between traits, with significance assessed at *p* ≤ 0.05 and *p* ≤ 0.01. Furthermore, we constructed a spider plot to check the genetic variability. Principal component analysis (PCA) was performed in XLSTAT to explore data variability, and the first two principal components, which captured the most significant variability, were used to construct a genotype vs. trait biplot. This biplot is particularly useful for visualizing complex relationships between genotypes and traits, allowing for the grouping of accessions based on their fatty acid composition and illustrating how the genotypes differed in their fatty acid profiles. A hierarchical clustering tree analysis (dendrogram) was also constructed using JMP software (version 15.2) to illustrate genetic relationships among genotypes.

## 3. Results

**Figure 2:**
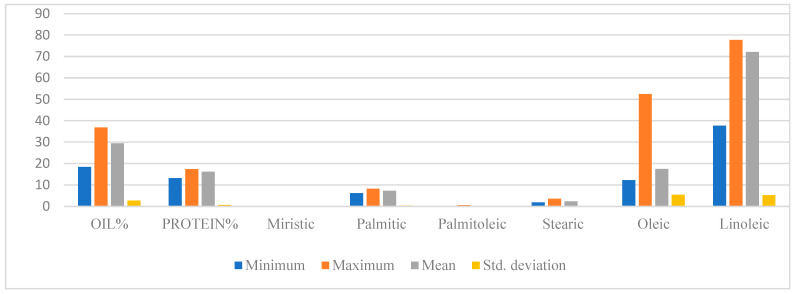
Graphical comparison of descriptive statistics for oil, protein, and fatty acids.

The analysis of variance (ANOVA) revealed significant differences among the genotypes for most traits except for myristic acid content (Table 2). A diverse array of variations in fatty acid composition was observed throughout the course of the study. The mean value of oil % was 29.49 while minimum and maximum were found in China 1 (18.44%) and Egypt 1 (36.88%). The maximum protein percentage was found in the genotype “Dincer” while the minimum was reported in “China 1”. Furthermore, among the different fatty acids analyzed, China 1 exhibited the highest concentration of myristic acid at 0.170%, while the lowest concentration, 0.100%, was observed in Remzibey; the palmitic acid content in Egypt 3 showed a maximum of 8.20%, contrasting with the minimum of 6.13% found in Bangladesh 3. For palmitoleic acid, the highest concentration, 0.53%, was found in Jordan 5, while the lowest, 0.03%, was noted in Bangladesh 2 and Portugal 2. Regarding stearic acid, Argentina 1 had the highest level at 3.51%, while Bangladesh 2 exhibited the lowest at 1.90%. The maximum oleic acid percentage recorded was 52.5% in China 7, whereas the minimum, 12.22%, was detected in Iran 1. The linoleic acid percentage was 77.73% in Iran 1, while China 7 had the lowest content at 37.7%. The average values of all examined fatty acids in 87 safflower accessions are presented in Table 3. The statistical analysis involved examining the mean, standard deviation, minimum, and maximum values of all traits, and tests were conducted to gain insights into the variations in the fatty acids in the safflower germplasm under study (Table 4 and Figure 2).

**Figure 3:**
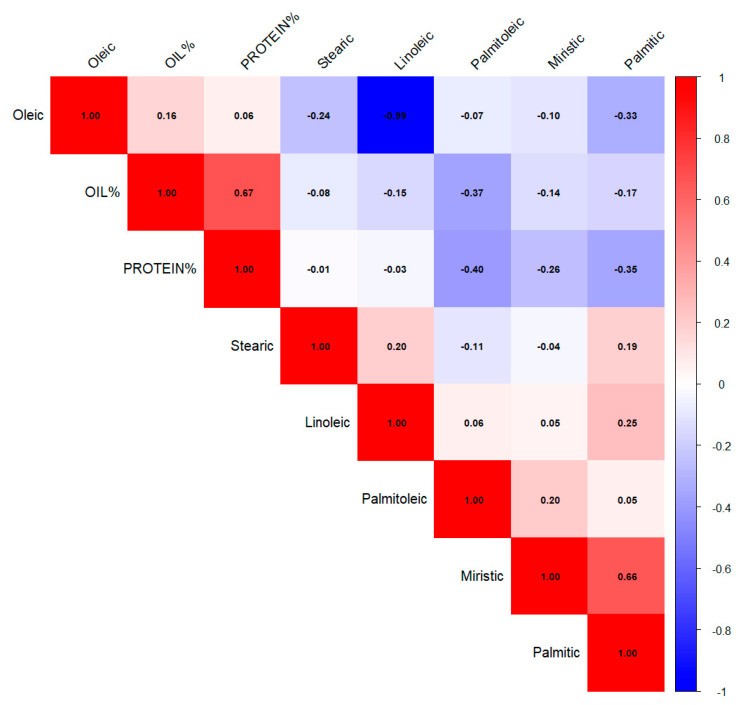
Correlation coefficients among oil, protein, and fatty acids in safflower accessions.

A correlation analysis was conducted to examine the relationship between the studied fatty acids. A highly significant correlation (*p* > 0.05) was observed among the various fatty acids studied, which enhances the statistical power of the tests. Only values above 0.05 will be discussed in this context. There was a strong and positive correlation (r = 0.673*) observed between protein and oil. Conversely, protein exhibited a highly negative correlation with myristic, palmitic, and palmitoleic acids, as indicated in Figure 3.

A highly significant and positive correlation was observed between “Palmitic acid and Myristic acid” (0.655*) and “Palmitic acid and Linolic acid” (0.251*). While a highly negative correlation was found among “Oleic and linoleic acid, Palmitoleic acid with Oil and Protein, Oleic acid with Palmitic acid and Stearic acid”. The correlation between “Oleic and Linoleic acid” was also found to be highly negative (−0.995*) (Figure 3). The spider plot illustrates the comparative variation in oil, protein, and fatty acid characteristics among safflower genotypes, normalized to the panel’s mean. Marked variability was noted, with particular genotypes displaying the highest (↑) and lowest (↓) values for each characteristic. China 7 exhibited a high oleic acid content (positive response), whereas Iran 1 displayed the lowest oleic acid value (negative response). In contrast, Iran 1 had superior performance in terms of linoleic acid, whereas China 7 recorded the lowest value.

**Figure 4:**
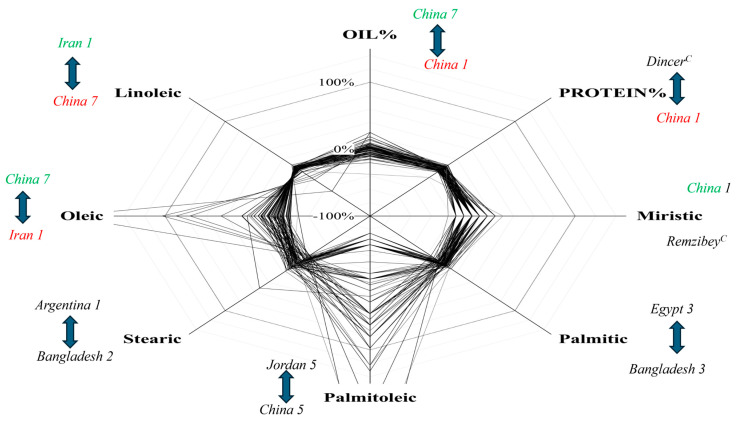
Relative variation among traits measured in 87 safflower genotypes. ^C^: Cultivar. The spider plot shows the percentage variation in oil and fatty acids for every line, normalized by the mean value of the panel. The genotype with the highest (↑) and lowest (↓) value for each trait of study is indicated in italics; if the genotype is repeated, it will appear in green (positive response) or red (negative response).

In terms of total oil percentage, China 7 exhibited the greatest value, whereas China 1 recorded the lowest. The protein content exhibited the most extensive variation, with the Dincer cultivar attaining the greatest value and China 1 the lowest. Traits such as stearic and palmitic acid exhibited significant disparities, with Argentina 1 and Bangladesh 2 demonstrating superiority in terms of their stearic acid contents, whereas Egypt 3 and Bangladesh 3 represented the extreme values for palmitic acid content (Figure 4). A selection of superior genotypes based on the desired traits was also carried out and these genotypes are presented in Table 5. The results of the study revealed a relatively high broad-sense heritability for all traits assessed. Notably, protein percentage exhibited a high heritability but a low genetic advance as a percentage of the mean (GAM). In contrast, palmitic acid, oil percentage, stearic acid, and linoleic acid showed a high heritability with moderate GAM. On the other hand, palmitoleic acid and oleic acid demonstrated both a high heritability and a high GAM, suggesting that there is a greater potential for genetic improvement in these traits (Table 6).

To explore the range of variation within the safflower germplasm under investigation, PCA was conducted on the fatty acids studied, utilizing a correlation matrix. We computed the eigenvalues associated with each principal component, which represent the amount of variability explained by each component. For clarity and interpretability, we focused on the principal components that together explained a substantial portion of the total variance in the data. Specifically, we retained the first five principal components (PCs) based on their cumulative contribution of 92.92% to the total observed variations, a threshold indicating that the major patterns of variation are effectively captured by these components (Table 7).

The first principal component (PC1) explained the highest proportion of the variance at 33.05%, with oleic acid identified as the main contributor to this component, representing the variation associated with oleic acid levels. The second component (PC2) accounted for 23.48% of the total variation, with linoleic acid identified as its primary contributor, capturing variations in linoleic acid levels. PC3 and PC4 contributed 16.09% and 12.28% of the total variance, respectively, with myristic and stearic acids as the main contributors, reflecting variations in these fatty acids. By selecting these components, we ensured that the primary sources of variability in the dataset were represented, providing insights into the relationship between fatty acid composition and safflower genotype characteristics.

**Figure 5:**
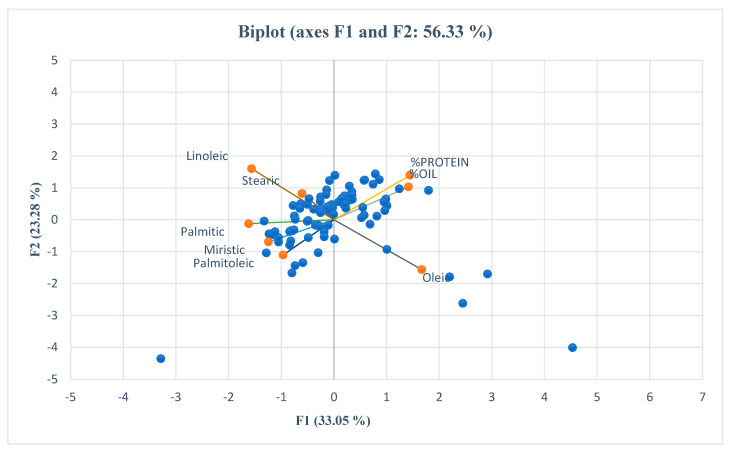
Biplot diagram of the main fatty acid components of the safflower accessions.

To further examine the patterns of variation among the studied materials, the first two principal components (PC1 and PC2), which captured the most significant variability, were used to construct a genotype vs. trait biplot (GT Biplot) (Figure 5). This biplot facilitated the grouping of accessions based on their fatty acid composition, including myristic, palmitic, palmitoleic, stearic, oleic, and linoleic acids, illustrating how the genotypes differed in their fatty acid profiles.

**Figure 6:**
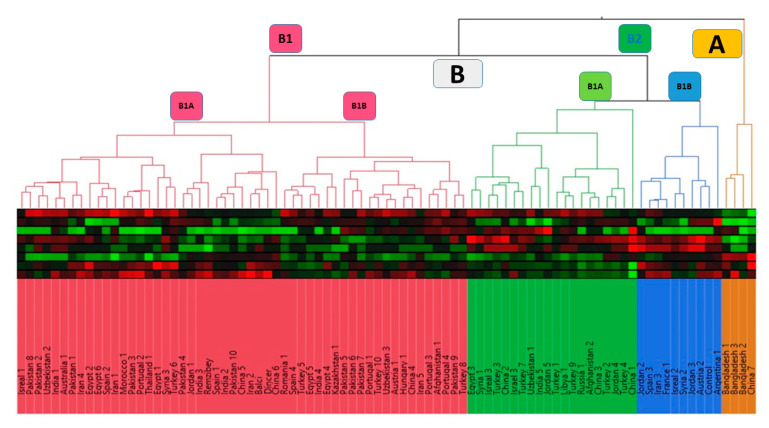
Dendrogram for oil, protein, and fatty acids using 87 safflower accessions.

In order to comprehend the correlation between fatty acids, a dendrogram was constructed (Figure 6). The dendrogram based on oil, protein, and fatty acid composition traits separated the 87 studied accessions into two populations. The first population consisted of four accessions, with all three accessions from Bangladesh clustered together in population A. The remaining accessions fell into population B, which was further divided into two sub-populations, B1 and B2. Population B1 consisted of 53 accessions, further classified into B1A and B1B with 31 and 22 accessions, respectively. Population B2 consisted of 30 accessions, further classified into B2A and B2B with 20 and 10 accessions, respectively.

## 4. Discussion

In agricultural experiments, the primary objective is to assess the adaptability of new plant germplasms to specific climate conditions and determine their potential yield and performance in each cultivation area. These evaluations determine farmers’ decisions regarding the incorporation of new germplasm into their cropping systems, to enhance variety selection and increase agricultural income. In this investigation, 87 safflower genotypes sourced from various origins were evaluated in an area traditionally dedicated to cereal cultivation, such as durum wheat and barley. Notably, substantial disparities were observed in the oil and fatty acid composition among the tested genotypes over the course of the study. These variations can be attributed to a combination of genetic and environmental factors. Importantly, the study ensured uniform climate conditions and cultivation practices across all safflower genotypes, thereby establishing that the observed differences in oil and fatty acid composition were primarily influenced by genotype responses to prevailing environmental conditions.

Vegetable oils play a crucial role in human nutrition and find extensive applications in various industries as well as cosmetics and biofuel production [46]. The utilization of oils across various domains depends significantly on their compositional attributes. The fatty acid composition of oils is intricately linked to the presence and functionality of enzymes involved in their biosynthesis [46]. Safflower, an emerging oilseed crop, has garnered attention for its superior oil quality and favorable agronomic characteristics, including tolerance to drought and cold, rendering it well-suited for Mediterranean climates [11]. The significance attributed to safflower primarily stems from the high-quality oil extracted from its seeds [22], with particular emphasis on the variability in fatty acid content. The growing interest in oilseed crops for agro-industrial research and development initiatives has been notable in the Mediterranean region in recent years [28].

Mediterranean farmers, predominantly reliant on monocultures of winter cereals, seek low-input and alternative winter crops to diversify their cropping systems and advance agricultural sustainability [7]. The diversification of cropping systems stands as a cornerstone of the agroecological transition, yet comprehensive local assessments regarding potential new species, notably alternative oilseed crops, are largely absent, particularly under real on-farm circumstances [7]. Safflower has emerged as a notable candidate among various crop alternatives due to its wide environmental adaptability, minimal input requirements, robust plant vigor, even in marginal soil conditions, and capacity to withstand low temperatures.

Numerous factors, including climatic conditions, plant variety, and geographic location, exert significant influence on the oil content of safflower seeds [47,48]. Previous research indicates a wide range of oil contents spanning from 14.0% to 48.3%, reflecting substantial variability [48]. This variability is largely attributable to the genetic potentials of safflower genotypes and their adaptive capacities. In our study, we observed the oil content ranging from 18.44% to 36.88%. Notably, Zemour et al. [22] documented oil content variations ranging from 15% to 40% among safflower genotypes, while Abou Chehade et al. [11] reported a range of 20.3% to 35.8%. The oil content of safflower seeds is predominantly influenced by genetic traits, environmental conditions, and agronomic practices, like other oilseed crops [7]. In research conducted by Zanetti et al. [7], the oil content of safflower genotypes exhibited a range spanning from 36.6% to 40.2%. Our own findings closely align with these previously reported results, particularly those documented in Mediterranean regions [11,28,49].

Additionally, studies in arid and semi-arid regions have also reported oil concentrations ranging from 17% to 43% in safflower genotypes [28,34,50]. However, the genetic inheritance pattern of seed oil content in safflower remains to be fully elucidated [51]. Our results demonstrate that safflower seeds exhibit a substantial oil content under semi-arid conditions, with the cultivar selection playing a critical role [52,53,54].

The protein content of safflower seeds serves as a significant metric for seed quality, particularly due to its valuable application in livestock feed. Previous studies by La Bella et al. [28] and Shahrokhnia and Sepaskhah [55] have documented protein contents ranging from 10% to 22%, a range consistent with the findings of the current investigation, which yielded protein contents between 13.17% and 17.36%. Despite this consistency, our study revealed an inconsistent protein content across different safflower genotypes. Such inconsistencies may stem from the contrasting responses of seed protein content to varying environmental conditions. However, it is noteworthy that genotype × environment interactions may contribute to diverse cultivar responses, given that protein concentration is a trait subject to quantitative inheritance [56].

The variability in fatty acid content within safflower seed oil makes this oleaginous species a subject of significant interest [28,57]. This composition is influenced by both the cultivars employed and the prevailing climatic conditions throughout the crop cycle [22]. Previous studies have substantiated this dependence [11,17,58,59]. Consequently, exploring the genetic diversity of safflower holds promise for germplasm conservation and utilization strategies, thereby enhancing breeding programs tailored to semi-arid regions to foster sustainable crop production [60,61]. The industrial value of vegetable oil is intricately tied to its fatty acid profile. Conventional safflower oil typically comprises 6–8% palmitic acid, 2–3% stearic acid, 16–20% oleic acid, and 71–75% linoleic acid [33].

Previous research has documented variations in the content of palmitic acid (PA) and stearic acid among different safflower genotypes. Johnson et al. [62] reported PA contents ranging from 3.9% to 6.8%, while Uysal et al. [63] and Zemour et al. [22] observed ranges of 6.0% to 8.5% and 6.6% to 7.15%, respectively. In our study, the PA content ranged from 6.13% to 8.2%, aligning with these previous findings. Similarly, the variability in stearic acid content reported by Johnson et al. [62] (1.1% to 4.5%) and Uysal et al. [63] (2.0% to 3.1%) corresponds closely to our observations.

Variations were also noted in linoleic acid ranging from 37.7% to 77.7% and oleic acid from 12.2% to 52.5%, consistent with the findings of Zemour et al. [22]. Oleic and linoleic acids are pivotal for safflower oil quality and human health. The predominance of these acids underscores their significance in characterizing the safflower germplasm, both agronomically and economically [28]. Safflower genotypes exhibiting elevated levels of oleic and linoleic acids represent valuable genetic resources for enhancing safflower oil quality in the Mediterranean region. Conventionally, safflower oil comprises primarily palmitic acid (4–8%), stearic acid (2–3%), oleic acid (8–18%), and linoleic acid (73–84%) [16]. Genotypes with a high oleic acid content, compared to linoleic acid, are deemed significant for oil quality owing to their influence on oil stability and human health.

The literature reports variations in both oleic and linoleic acid among safflower genotypes. For instance, Murthy and Anjani [64] explored the seed oil compositions of seven Carthamus species from Pakistan, revealing oleic acid and linoleic acid ranges of 11.3–23.4% and 61.4–82.1%, respectively. Similarly, Sabzalian et al. [65] noted oleic acid levels between 12.2% and 19.8%, and linoleic acid levels ranging from 62.5% to 76.1% across different genotypes. Kurt et al. [39] reported oleic acid content varying from 7.14% to 14.05% and linoleic acid content ranging from 74.65% to 84.13% among various safflower genotypes.

In previous research, the oleic acid content fluctuated between 6.2% and 81.9% [62], 62.7% and 82.1% [66], and 7.8% and 30.6% [63]. Similarly, the linoleic acid content ranged from 11.0% to 83.1% [62], 70.3% to 78.8% [66], and 60.0% to 81.6% [63]. The findings of this study corroborate previously reported data, particularly highlighting the economic significance of safflower oil with high oleic acid content (>75%) due to its enhanced oxidative stability, compared to typical safflower oil rich in polyunsaturated fatty acids [67,68].

The spider plot demonstrates the genetic potential of safflower genotypes for enhancing critical attributes, including oil and protein content, along with fatty acid profiles. Genotypes like China 7, which consistently demonstrate elevated oleic acid and total oil percentages, are optimal selections for breeding efforts aimed at enhancing oil stability and health benefits. In contrast, genotypes such as Iran 1, which are rich in linoleic acid, present prospects for nutritional markets focused on polyunsaturated fats. The negative correlation observed between oleic and linoleic acids reflects their metabolic trade-off, suggesting the need for a balanced selection approach when targeting both stability and nutritional quality. Similarly, the variability in traits like protein content and saturated fatty acids (e.g., stearic and palmitic acids) demonstrates the complexity of optimizing multiple traits simultaneously. Spider plots provide visual clarity for identifying superior genotypes and potential outliers, aiding breeders in making informed decisions and developing cultivars for industrial, nutritional, and environmental demands.

Heritability and variance components play a pivotal role in designing effective breeding programs, predicting responses to selection, and constructing selection indices. In this study, high heritability was observed for oil content and fatty acids, indicating that these traits are largely governed by genetic factors and minimally influenced by environmental conditions. This supports the feasibility of selection based on phenotypic observations of these traits.

A high heritability combined with a low GAM for protein percentage suggests a greater role of environmental factors rather than genetic components, making selection less effective for this trait. Conversely, traits such as oil percentage, palmitic acid, stearic acid, and linoleic acid exhibited a high heritability with moderate GAM, indicating the involvement of both additive and non-additive gene actions. These traits may benefit from population improvement strategies such as reciprocal recurrent selection. The combination of high heritability and high GAM observed for palmitoleic acid and oleic acid is particularly significant. This suggests strong genetic control and substantial potential for genetic improvement through selection, making these traits ideal targets in breeding programs. Consistent with these findings, prior research has reported high heritability values for oil content (67.6%) and fatty acids, with oleic acid and linoleic acid showing a heritability of 99.4% and 99.3%, respectively [69]. This highlights the potential for selective breeding to enhance the oil yield and fatty acid profile of crops like safflower, as demonstrated in studies by Golkar and Karimi [70]. Collectively, these findings emphasize the importance of leveraging traits with a high heritability and GAM in breeding programs, particularly for improving oil quality and productivity in oilseed crops.

High-oleic-content safflower oil is gaining prominence, particularly among health-conscious consumers [51]. A notable negative correlation between oleic and linoleic acid levels has been observed [71], consistent with the findings of this investigation. This correlation alongside reports of an insignificant relationship between oil content and individual fatty acid levels [72], underscores the feasibility of breeding high-oleic-content safflower varieties with elevated oil contents [43,71,73,74]. Similar trends have been noted in high-oleic-content sunflowers, where no significant association between oleic acid and oil content was observed, but a significant negative correlation between oleic acid and linoleic acid was present. This trend aligns with previous findings, where oleic and linoleic acid concentrations exhibit an inverse relationship due to their shared biosynthetic pathway. In safflower, the enzyme Δ12 desaturase catalyzes the conversion of oleic acid (18:1) to linoleic acid (18:2). Thus, higher Δ12 desaturase activity leads to increased linoleic acid levels and reduced oleic acid levels, explaining the observed inverse relationship. This balance between oleic and linoleic acid content is of agronomic significance, as higher oleic acid levels contribute to oil stability, while a higher linoleic acid content enhances nutritional value [51,71,73,75,76,77].

As can be seen in the dendrogram (Figure 6), the accessions in group A and those in group B have similar values except for oleic and linoleic acid content. The four accessions in group A exhibit the highest oleic acid and lowest linoleic acid values, distinguishing them from the other groups. These findings have the potential to enhance knowledge of cultivar responses under Mediterranean conditions and offer valuable insights for breeders regarding their integration into selection programs. Additionally, the results serve to identify promising safflower genotypes suitable for cultivation in the Mediterranean region and for incorporation into future breeding initiatives.

While the findings of this study generally align with previous research on safflower genotypes, some observed differences in fatty acid profiles, including oleic, linoleic, and other fatty acids, may stem from variations in environmental conditions, genetic diversity, and methodological approaches. Environmental factors such as temperature and rainfall, particularly within the Mediterranean climate of Adana, Türkiye, are known to influence the biosynthesis of fatty acids in oilseed crops. Such climatic conditions may partly explain the observed variations when compared to studies conducted in other regions with different environmental profiles. Additionally, this study employed Soxhlet extraction for oil analysis and was conducted over a single growing season, while other studies may have used different extraction methods (e.g., cold pressing or supercritical CO₂ extraction) or spanned multiple growing seasons. These methodological and environmental factors are crucial to consider when interpreting comparative results, as they may impact the phenotypic expression of various fatty acid traits, influencing the overall oil content and composition in safflower genotypes. The data from this study provide actionable insights for breeding programs focused on improving safflower oil quality and yield. Specifically, accessions with a higher oleic acid content could be prioritized in breeding programs for enhanced oil stability and shelf life, which is particularly beneficial for industrial oil applications. Conversely, genotypes with elevated linoleic acid levels could be targeted for nutritional breeding programs, given linoleic acid’s recognized health benefits. By leveraging the observed genetic variability in fatty acid profiles, breeders can select parent lines with complementary traits to enhance genetic diversity and achieve desirable trait combinations in progeny. These findings inform breeding strategies to develop safflower varieties tailored to meet specific industrial or nutritional standards, thus aligning breeding goals with market demands.

**Selection of Superior Genotypes**

One of the main objectives of this study was the identification and selection of high-performing safflower genotypes based on desirable traits. Safflower oil, the primary product of this crop, is valued for its high oil and protein content, which are beneficial for human nutrition. The selection process focused on genotypes with superior traits, such as increased oil and protein content, coupled with reduced concentrations of palmitic and myristic acids. These fatty acids, when present in lower concentrations, contribute to the nutritional value of the oil.

Among the evaluated accessions, *Egypt 1*, *China 7*, *Turkey 6*, *Egypt 2*, *Syria 3*, *Jordan 2*, *Iran 2*, *Portugal 2*, *Egypt 4*, and *Remzibey* demonstrated superior performances, exhibiting higher oil and protein percentages. This is further supported by the strong positive correlation observed between oil and protein content. In terms of fatty acid composition, these accessions also showed low levels of myristic and palmitic acids, aligning with the selection criteria.

To ensure the robustness of these findings, multi-year and multi-location yield trials are currently underway to validate the performance of the selected genotypes. Following successful characterization and evaluation, the superior accessions will be submitted for registration with the Seed Registration and Certification Center of the Agriculture and Forestry Ministry of Turkey. These characterized accessions are available for use by the safflower breeding community under the jurisdiction of the Turkish Seed Transfer Act, facilitating further breeding and research initiatives.

## 5. Conclusions

In conclusion, this study has provided valuable insights into the oil content and fatty acid composition of the safflower (*Carthamus tinctorius* L.) germplasm. Significant variations were observed among the safflower accessions, with genotype Egypt 1 displaying the highest oil content. The correlation analysis revealed strong associations between protein and oil content, as well as between certain fatty acids. These findings underscore the importance of characterizing safflower germplasm diversity for developing cultivars with improved oil quality and nutritional value. Such advancements hold promise for enhancing sustainable production and meeting the diverse needs of the food, pharmaceutical, and industrial sectors. Further research and breeding efforts focused on exploiting the genetic diversity within safflower germplasm can contribute to the development of high-yielding and nutritionally rich cultivars, thereby advancing the utilization and commercialization of safflower as a valuable oilseed crop.

## Figures and Tables

**Table 1:** Passport data of 87 safflower accessions collected from 11 different countries.

Sr. No	Genotype Code	Genotype Name	Sr. No	Genotype Code	Genotype Name
**1**	P1-198990	Isreal 1	**45**	P1-314650	Kazakhstan 1
**2**	P1-209287	Romania 1	**46**	P1-340086	Turkey 5
**3**	P1-239042	Morocco 1	**47**	P1-367833	Argentina 1
**4**	P1-250082	Egypt 1	**48**	P1-369846	Uzbekistan 2
**5**	P1-250194	Pakistan 1	**49**	P1-369853	Uzbekistan 3
**6**	P1-250201	Pakistan 2	**50**	P1-386174	Syria 3
**7**	P1-250345	Pakistan 3	**51**	P1-387821	Thailand 1
**8**	P1-250346	Pakistan 4	**52**	P1-405958	Iran 2
**9**	P1-250351	Pakistan 5	**53**	P1-405967	Iran 3
**10**	P1-250353	Pakistan 6	**54**	PI-401478	Bangladesh 1
**11**	P1-250481	Pakistan 7	**55**	PI-401480	Bangladesh 2
**12**	P1-250528	Egypt 2	**56**	PI-199878	India 5
**13**	P1-250532	Egypt 3	**57**	PI-220647	Afghanistan 2
**14**	P1-250540	Egypt 4	**58**	PI-235660	Australia 1
**15**	P1-250601	India 1	**59**	PI-237538	Turkey 6
**16**	P1-250605	Egypt 5	**60**	PI-250474	Pakistan 8
**17**	P1-250608	Egypt 6	**61**	PI-250478	Pakistan 9
**18**	P1-250720	Iran 1	**62**	PI-250840	Iran 4
**19**	P1-251284	Jordan 1	**63**	PI-251265	Jordan 3
**20**	P1-251285	Jordan 2	**64**	PI-251267	Jordan 4
**21**	P1-253386	Isreal 2	**65**	PI-251268	Jordan 5
**22**	P1-253388	Spain 1	**66**	PI-251290	Israel 3
**23**	P1-253391	Spain 2	**67**	PI-251978	Turkey 7
**24**	P1-253394	Spain 3	**68**	PI-251984	Turkey 8
**25**	P1-253395	Spain 4	**69**	PI-253519	Austria 1
**26**	P1-253553	Portugal 1	**70**	PI-288983	Hungary 1
**27**	P1-253556	Portugal 2	**71**	PI-393499	Libya 1
**28**	P1-253564	Portugal 3	**72**	PI-401470	Bangladesh 3
**29**	P1-253764	Afghanistan 1	**73**	PI-406010	Iran 5
**30**	P1-253571	Portugal 4	**74**	PI-406701	Turkey 9
**31**	P1-253892	Isreal 3	**75**	PI-406702	Turkey 10
**32**	P1-253900	Syria 1	**76**	PI-426521	Pakistan 10
**33**	P1-253898	Syria 2	**77**	PI-543979	China 3
**34**	P1-262435	Uzbekistan 1	**78**	PI-543982	China 4
**35**	P1-262452	China 1	**79**	PI-544001	China 5
**36**	P1-262453	China 2	**80**	PI-568809	China 6
**37**	P1-304498	Turkey 1	**81**	PI-568874	China 7
**38**	P1-304502	Turkey 2	**82**	PI 576985	France 1
**39**	P1-304504	Turkey 3	**83**	BVAL-901352	Austria 2
**40**	P1-304505	Turkey 4	**84**	Control	Control
**41**	P1-305195	India 2	**85**	Remzibey	Remzibey ^C^
**42**	P1-305535	Russia 1	**86**	Dincer	Dincer ^C^
**43**	P1-306941	India 3	**87**	Balci	Balci ^C^
**44**	P1-306976	India 4			

^C^: Cultivar.

**Table 2:** Analysis of variance in the studied traits for various safflower germplasms.

Source	df	Mean Squares							
		Oil (%)	Protein (%)	Miristic	Palmitic	Palmitoleic	Stearic	Oleic	Linoleic
Treatment (ignoring Blocks)	86	**	**	ns	**	**	**	**	**
Treatment: Check	3	**	**	ns	**	ns	**	**	**
Treatment: Test	82	**	**	ns	**	**	**	**	**
Treatment: Test vs. Check	1	**	**	ns	**	**	**	**	**
Block (eliminating Treatments)	4	ns	ns	ns	ns	ns	ns	ns	ns
Residuals	12								

**: Significant, ns: non significant.

**Table 3:** Oil, protein content, and fatty acid composition for safflower accessions.

Genotypes	Oil (%)	Protein (%)	Miristic	Palmitic	Palmitoleic	Stearic	Oleic	Linoleic
Isreal 1	28.46 ± 0.479	16.31 ± 0.34	0.13 ± 0.002	7.45 ± 0.28	0.05 ± 0.02	2.41 ± 0.05	17.39 ± 0.388	71.76 ± 1.9
Romania 1	27.78 ± 0.617	16.26 ± 0.402	0.12 ± 0.004	6.81 ± 0.25	0.04 ± 0.01	2.35 ± 0.04	15.03 ± 0.16	75.63 ± 1.94
Morocco 1	30.09 ± 0.838	16.92 ± 0.466	0.12 ± 0.005	6.91 ± 0.28	0.04 ± 0.01	2.33 ± 0.05	15.06 ± 0.14	75.53 ± 2.05
Egypt 1	36.89 ± 0.865	16.22 ± 0.454	0.12 ± 0.005	7.17 ± 0.3	0.04 ± 0.01	2.25 ± 0.06	15.81 ± 0.25	74.2 ± 2.24
Pakistan 1	31.87 ± 0.438	16.37 ± 0.542	0.13 ± 0.003	7.51 ± 0.29	0.05 ± 0.02	2.17 ± 0.03	16.63 ± 0.15	73.33 ± 1.29
Pakistan 2	30.42 ± 0.553	15.55 ± 1.529	0.14 ± 0.008	7.58 ± 0.26	0.05 ± 0.02	2.38 ± 0.05	13.58 ± 0.33	76.1 ± 1.42
Pakistan 3	31.59 ± 0.521	17 ± 0.531	0.13 ± 0.002	6.99 ± 0.27	0.04 ± 0.01	2.33 ± 0.04	15.07 ± 0.3	74.75 ± 2.06
Pakistan 4	29.12 ± 0.469	16.18 ± 0.345	0.11 ± 0.004	7.01 ± 0.17	0.11 ± 0.02	2.53 ± 0.04	16.52 ± 0.34	73.22 ± 1.44
Pakistan 5	26.67 ± 0.804	15.65 ± 0.582	0.11 ± 0.003	6.82 ± 0.17	0.26 ± 0.03	2.42 ± 0.04	18.71 ± 0.25	71.58 ± 1.61
Pakistan 6	30.13 ± 0.799	16.54 ± 0.408	0.13 ± 0.002	6.56 ± 0.18	0.19 ± 0.01	2.42 ± 0.03	18.19 ± 0.31	71.63 ± 1.59
Pakistan 7	30.52 ± 0.908	15.89 ± 0.676	0.12 ± 0.004	6.58 ± 0.16	0.18 ± 0.03	2.44 ± 0.03	14.86 ± 0.4	74.97 ± 1.73
Egypt 2	34.92 ± 0.846	16.66 ± 0.432	0.13 ± 0.002	7.56 ± 0.33	0.11 ± 0.03	1.93 ± 0.031	14.32 ± 0.34	75.24 ± 1.16
Egypt 3	27.57 ± 0.459	16.1 ± 0.273	0.14 ± 0.008	8.2 ± 0.28	0.17 ± 0.02	2.31 ± 0.011	13.74 ± 0.28	74.96 ± 1.19
Egypt 4	32.11 ± 0.235	15.78 ± 0.415	0.12 ± 0.004	7.22 ± 0.35	0.1 ± 0.01	2.28 ± 0.017	14.94 ± 0.28	74.59 ± 1.51
India 1	28.97 ± 0.897	16.71 ± 0.57	0.14 ± 0.005	7.52 ± 0.23	0.15 ± 0.01	2.32 ± 0.012	13.99 ± 0.36	75 ± 1.31
Egypt 5	25.27 ± 0.391	15.79 ± 0.555	0.12 ± 0.004	7.28 ± 0.27	0.11 ± 0.01	2.46 ± 0.012	15.85 ± 0.2	73.44 ± 0.56
Egypt 6	30.84 ± 0.879	16.3 ± 0.327	0.13 ± 0.002	7.31 ± 0.23	0.12 ± 0.02	1.97 ± 0.013	14.21 ± 0.32	75.53 ± 1.25
Iran 1	30.57 ± 9.303	16.41 ± 0.33	0.12 ± 0.004	7.46 ± 0.14	0.17 ± 0.01	2.01 ± 0.012	12.22 ± 0.36	77.73 ± 1.77
Jordan 1	29.91 ± 1.018	16.41 ± 0.306	0.11 ± 0.004	6.71 ± 0.23	0.05 ± 0.02	2.49 ± 0.014	19.09 ± 0.2	71.29 ± 1.21
Jordan 2	33.88 ± 0.864	16.17 ± 0.354	0.16 ± 0.006	7.76 ± 0.18	0.12 ± 0.03	2.05 ± 0.016	17.01 ± 0.22	72.21 ± 0.65
Isreal 2	29.15 ± 0.384	15.73 ± 0.467	0.14 ± 0.008	7.46 ± 0.18	0.05 ± 0.01	2.17 ± 0.025	18.21 ± 0.3	71.26 ± 1.15
Spain 1	29.15 ± 0.519	16.42 ± 0.387	0.12 ± 0.004	6.97 ± 0.18	0.04 ± 0.01	2.08 ± 0.016	19.17 ± 0.35	70.92 ± 1.04
Spain 2	30.69 ± 0.476	16.51 ± 0.473	0.13 ± 0.003	7.05 ± 0.23	0.06 ± 0.02	2.02 ± 0.015	14.93 ± 0.26	75.14 ± 1.11
Spain 3	31.56 ± 0.632	16.71 ± 0.465	0.15 ± 0.009	7.54 ± 0.19	0.05 ± 0.02	2.16 ± 0.006	16.03 ± 0.17	73.39 ± 0.74
Spain 4	28.05 ± 0.336	15.89 ± 0.531	0.13 ± 0.003	7.04 ± 0.16	0.04 ± 0.01	2.41 ± 0.011	15.93 ± 0.26	74.21 ± 0.65
Portugal 1	28.32 ± 0.435	16.6 ± 0.406	0.13 ± 0.002	6.95 ± 0.26	0.1 ± 0.01	2.27 ± 0.012	16.4 ± 0.31	73.49 ± 0.63
Portugal 2	32.35 ± 0.384	17.09 ± 0.417	0.13 ± 0.003	6.83 ± 0.2	0.03 ± 0.01	2.55 ± 0.009	16.2 ± 0.32	74.03 ± 0.99
Portugal 3	29.09 ± 0.357	15.58 ± 0.361	0.12 ± 0.004	7.29 ± 0.26	0.21 ± 0.04	2.5 ± 0.1	15.38 ± 0.42	73.59 ± 0.59
Afghanistan 1	28.23 ± 0.397	15.8 ± 0.54	0.13 ± 0.006	7.32 ± 0.27	0.2 ± 0.01	2.69 ± 0.011	14.88 ± 0.81	73.8 ± 0.8
Portugal 4	29.55 ± 0.472	15.2 ± 0.42	0.13 ± 0.002	7.13 ± 0.25	0.27 ± 0.03	2.44 ± 0.012	14.75 ± 0.52	75.21 ± 0.65
Isreal 3	29.06 ± 0.289	15.54 ± 0.375	0.15 ± 0.009	7.6 ± 0.14	0.21 ± 0.05	2.11 ± 0.011	15.13 ± 0.26	74.23 ± 0.75
Syria 1	28.07 ± 0.682	15.95 ± 0.428	0.13 ± 0.007	7.86 ± 0.13	0.11 ± 0.03	2.12 ± 0.016	14.37 ± 0.5	74.6 ± 0.6
Syria 2	30.12 ± 0.332	15.83 ± 0.469	0.15 ± 0.008	7.58 ± 0.16	0.04 ± 0.01	1.97 ± 0.005	18.59 ± 0.35	71.33 ± 0.46
Uzbekistan 1	27.28 ± 0.398	15.52 ± 0.278	0.13 ± 0.006	7.56 ± 0.15	0.23 ± 0.04	1.93 ± 0.013	15.29 ± 0.14	74.66 ± 0.87
China 1	18.44 ± 0.58	13.17 ± 0.39	0.17 ± 0.005	7.98 ± 0.16	0.45 ± 0.04	2.3 ± 0.013	20.43 ± 0.27	68.1 ± 1.1
China 2	30.35 ± 0.436	15.99 ± 0.506	0.15 ± 0.007	8.03 ± 0.18	0.26 ± 0.01	2.3 ± 0.018	15.6 ± 0.22	73.3 ± 0.89
Turkey 1	28.42 ± 0.539	15.61 ± 0.347	0.13 ± 0.002	7.24 ± 0.18	0.11 ± 0.02	2.19 ± 0.012	15.73 ± 0.17	73.84 ± 0.84
Turkey 2	26.45 ± 0.516	15.26 ± 0.241	0.13 ± 0.003	7.66 ± 0.24	0.05 ± 0.01	2.47 ± 0.014	21.98 ± 0.27	67.16 ± 0.68
Turkey 3	27.16 ± 0.375	15.32 ± 0.329	0.15 ± 0.007	7.87 ± 0.24	0.17 ± 0.03	2.09 ± 0.015	16.2 ± 0.2	73.17 ± 0.71
Turkey 4	27.43 ± 0.79	15.43 ± 0.368	0.14 ± 0.005	7.69 ± 0.23	0.05 ± 0.03	2.45 ± 0.014	17.25 ± 0.38	72.02 ± 0.96
India 2	29.67 ± 0.66	16.36 ± 0.384	0.12 ± 0.004	7.11 ± 0.24	0.05 ± 0.01	2.26 ± 0.016	18.09 ± 0.26	72.02 ± 1.14
Russia 1	23.5 ± 0.651	15.03 ± 0.406	0.13 ± 0.006	7.52 ± 0.16	0.05 ± 0.02	2.42 ± 0.012	14.88 ± 0.17	74.66 ± 0.69
India 3	31.32 ± 0.394	16.76 ± 0.527	0.11 ± 0.004	7.29 ± 0.17	0.04 ± 0.01	2.49 ± 0.012	17.25 ± 0.25	72.07 ± 1.05
India 4	26.65 ± 0.615	15.6 ± 0.317	0.12 ± 0.004	7.27 ± 0.14	0.04 ± 0.01	2.35 ± 0.012	18.15 ± 0.19	72.03 ± 1.05
Kazakhstan 1	29.16 ± 0.39	15.17 ± 0.384	0.11 ± 0.004	7.39 ± 0.13	0.04 ± 0.01	2.39 ± 0.014	15.14 ± 0.36	74.26 ± 1.2
Turkey 5	26.17 ± 0.483	15.82 ± 0.471	0.13 ± 0.006	6.99 ± 0.17	0.11 ± 0.02	2.47 ± 0.012	17.33 ± 0.29	72.57 ± 0.71
Argentina 1	30.02 ± 0.581	16.77 ± 0.359	0.14 ± 0.008	7.68 ± 0.16	0.14 ± 0.01	3.51 ± 0.011	21.04 ± 0.946	67.29 ± 0.64
Uzbekistan 2	29.64 ± 0.66	16 ± 0.339	0.13 ± 0.006	7.58 ± 0.1	0.05 ± 0.01	2.4 ± 0.013	13.85 ± 0.29	75.18 ± 0.62
Uzbekistan 3	28.75 ± 1.713	16.16 ± 0.342	0.13 ± 0.003	7.14 ± 0.22	0.17 ± 0.02	2.33 ± 0.013	14.1 ± 0.31	75.69 ± 0.83
Syria 3	33.98 ± 0.864	16.71 ± 0.333	0.14 ± 0.004	7.68 ± 0.21	0.13 ± 0.02	2.33 ± 0.018	14.22 ± 0.1	74.81 ± 0.95
Thailand 1	31.81 ± 1.728	16.44 ± 0.36	0.13 ± 0.006	7.1 ± 0.18	0.04 ± 0.01	2.44 ± 0.016	12.4 ± 0.19	77.12 ± 1.12
Iran 2	33.36 ± 0.589	17.26 ± 0.333	0.13 ± 0.004	6.92 ± 0.13	0.04 ± 0.02	2.23 ± 0.011	18.7 ± 0.18	71.43 ± 0.98
Iran 3	30.99 ± 0.869	16.6 ± 0.429	0.15 ± 0.007	7.47 ± 0.11	0.04 ± 0.01	2.29 ± 0.017	16.54 ± 0.12	72.94 ± 0.89
Bangladesh 1	31.94 ± 0.661	16.11 ± 0.281	0.13 ± 0.006	6.54 ± 0.09	0.19 ± 0.01	1.92 ± 0.016	35.57 ± 0.09	55.2 ± 1.11
Bangladesh 2	28.14 ± 0.622	15.74 ± 0.401	0.11 ± 0.005	6.4 ± 0.08	0.03 ± 0.02	1.9 ± 0.002	30.79 ± 0.23	60.17 ± 1.17
India 5	26.73 ± 0.838	15.5 ± 0.324	0.13 ± 0.006	7.1 ± 0.21	0.34 ± 0.03	2.08 ± 0.016	17.61 ± 0.16	71.84 ± 0.84
Afghanistan 2	26.53 ± 0.614	15.31 ± 0.444	0.13 ± 0.003	7.47 ± 0.11	0.11 ± 0.01	2.3 ± 0.016	15.33 ± 0.25	74.03 ± 0.94
Australia 1	28.97 ± 0.804	16.33 ± 0.447	0.14 ± 0.005	7.57 ± 0.09	0.1 ± 0.01	2.4 ± 0.006	13.93 ± 0.25	75.11 ± 1.11
Turkey 6	34.99 ± 0.627	16.38 ± 0.407	0.13 ± 0.004	7.37 ± 0.09	0.17 ± 0.01	2.62 ± 0.006	13.66 ± 0.25	75.77 ± 0.88
Pakistan 8	28.92 ± 0.511	16.18 ± 0.347	0.12 ± 0.004	7.35 ± 0.06	0.05 ± 0.02	2.31 ± 0.005	13.9 ± 0.19	75.77 ± 0.77
Pakistan 9	29.75 ± 0.754	16.28 ± 0.41	0.12 ± 0.006	7.68 ± 0.12	0.19 ± 0.01	2.51 ± 0.009	17.04 ± 0.23	72.32 ± 0.64
Iran 4	31.59 ± 0.706	16.85 ± 0.467	0.13 ± 0.006	7.41 ± 0.12	0.04 ± 0.01	2.34 ± 0.008	15.42 ± 0.36	73.93 ± 0.93
Jordan 3	27.99 ± 0.861	15.5 ± 0.303	0.16 ± 0.006	7.88 ± 0.12	0.05 ± 0.01	2.58 ± 0.306	17.45 ± 0.43	71.61 ± 0.58
Jordan 4	25.19 ± 0.717	15.07 ± 0.449	0.14 ± 0.005	7.73 ± 0.06	0.08 ± 0.01	2.43 ± 0.011	21.91 ± 0.42	67.7 ± 0.82
Jordan 5	26.78 ± 0.772	16.03 ± 0.241	0.14 ± 0.008	6.91 ± 0.11	0.53 ± 0.02	1.91 ± 0.01	16.85 ± 0.2	73.66 ± 0.87
Israel 3	27.49 ± 0.663	16.55 ± 0.463	0.15 ± 0.008	7.38 ± 0.17	0.21 ± 0.01	2.07 ± 0.012	16.77 ± 0.23	72.57 ± 0.57
Turkey 7	27.52 ± 0.603	15.98 ± 0.338	0.14 ± 0.006	7.64 ± 0.15	0.23 ± 0.01	2.21 ± 0.008	16.49 ± 0.25	72.61 ± 0.76
Turkey 8	30.27 ± 0.543	16.28 ± 0.446	0.12 ± 0.006	7.25 ± 0.16	0.21 ± 0.01	2.56 ± 0.01	16.23 ± 0.23	72.91 ± 0.93
Austria 1	28.33 ± 0.669	16.45 ± 0.368	0.13 ± 0.006	7.07 ± 0.15	0.15 ± 0.02	2.24 ± 0.002	14.85 ± 0.37	74.7 ± 0.7
Hungary 1	30.97 ± 0.688	16.52 ± 0.488	0.12 ± 0.007	7.2 ± 0.12	0.17 ± 0.02	2.3 ± 0.009	14.87 ± 0.28	74.59 ± 0.72
Libya 1	27.17 ± 0.539	15.32 ± 0.402	0.14 ± 0.005	7.33 ± 0.12	0.19 ± 0.01	2.23 ± 0.009	14.37 ± 0.26	75.12 ± 1.12
Bangladesh 3	29.06 ± 0.591	17.07 ± 0.381	0.11 ± 0.005	6.13 ± 0.12	0.17 ± 0.01	1.94 ± 0.008	33.22 ± 0.01	57.85 ± 0.87
Iran 5	30.42 ± 0.693	16.28 ± 0.411	0.12 ± 0.006	7.02 ± 0.08	0.15 ± 0.02	2.34 ± 0.006	20.42 ± 0.16	69.23 ± 1.23
Turkey 9	28.07 ± 0.376	15.71 ± 0.388	0.14 ± 0.008	7.48 ± 0.12	0.15 ± 0.01	2.33 ± 0.006	15.88 ± 0.23	73.55 ± 0.76
Turkey 10	30.53 ± 0.703	16.41 ± 0.431	0.13 ± 0.006	6.93 ± 0.11	0.13 ± 0.01	2.3 ± 0.007	15.58 ± 0.2	74.21 ± 1.14
Pakistan 10	29.47 ± 0.717	16.57 ± 0.402	0.13 ± 0.003	7.26 ± 0.1	0.05 ± 0.02	2.08 ± 0.01	18.64 ± 0.15	70.66 ± 0.85
China 3	24.84 ± 0.772	15.35 ± 0.474	0.13 ± 0.004	7.6 ± 0.12	0.11 ± 0.01	2.44 ± 0.009	15.09 ± 0.22	73.99 ± 0.99
China 4	29.87 ± 0.754	16.76 ± 0.467	0.12 ± 0.007	7.17 ± 0.25	0.11 ± 0.01	2.35 ± 0.005	16.04 ± 0.16	73.79 ± 0.71
China 5	27.79 ± 0.774	16.93 ± 0.48	0.12 ± 0.005	6.7 ± 0.14	0.03 ± 0.01	2.25 ± 0.011	19.15 ± 0.15	70.98 ± 0.98
China 6	30.98 ± 0.618	16.1 ± 0.429	0.13 ± 0.006	7.48 ± 0.13	0.04 ± 0.01	2.15 ± 0.01	25.5 ± 0.5	63.5 ± 0.72
China 7	36.8 ± 0.67	16.39 ± 0.469	0.13 ± 0.003	7.03 ± 0.15	0.05 ± 0.01	1.97 ± 0.008	52.5 ± 0.7	37.7 ± 0.7
France 1	30.26 ± 0.555	16.7 ± 0.456	0.15 ± 0.008	7.59 ± 0.17	0.04 ± 0.02	2.2 ± 0.006	17.71 ± 0.2	71.43 ± 0.76
Austria 2	31.87 ± 0.651	16.51 ± 0.429	0.16 ± 0.006	8.15 ± 0.19	0.05 ± 0.01	2.64 ± 0.01	17.33 ± 0.25	70.67 ± 10.81
Control	30.29 ± 0.583	16.55 ± 0.401	0.14 ± 0.008	7.77 ± 0.12	0.05 ± 0.01	2.64 ± 0.006	18.41 ± 0.29	70.15 ± 11.15
Remzibey ^C^	31.99 ± 0.629	16.96 ± 0.444	0.1 ± 0.005	6.61 ± 0.13	0.05 ± 0.01	2.39 ± 0.005	20.73 ± 0.24	69.46 ± 0.83
Dincer ^C^	31.23 ± 0.662	17.36 ± 0.482	0.13 ± 0.006	7.35 ± 0.13	0.05 ± 0.01	2.17 ± 0.006	19.9 ± 0.19	70.14 ± 1.14
Balci ^C^	31.9 ± 0.68	16.8 ± 0.43	0.14 ± 0.005	6.83 ± 0.15	0.05 ± 0.01	2.21 ± 0.01	19.84 ± 0.16	70.14 ± 1.06

^C^: Cultivar.

**Table 4:** Maximum, minimum, mean and standard deviation (Std) for 8 different traits determined in the studied germplasm.

	Oil (%)	Protein (%)	Miristic	Palmitic	Palmitoleic	Stearic	Oleic	Linoleic
**Maximum**	36.885	17.362	0.17	8.2	0.53	3.51	52.5	77.73
**Minimum**	18.44	13.17	0.1	6.13	0.03	1.9	12.22	37.7
**Mean**	29.496	16.136	0.131	7.294	0.11	2.302	17.533	72.061
**Std**	2.735	0.634	0.013	0.399	0.09	0.23	5.396	5.235

**Table 5:** Most superior genotypes based on desired traits among the studied germplasms.

Genotypes	Oil (%)	Protein (%)	Myristic	Palmitic	Palmitoleic	Stearic	Oleic	Linoleic
Egypt 1	36.89	16.22	0.12	7.17	0.04	2.25	15.81	74.2
China 7	36.8	16.39	0.13	7.03	0.05	1.97	52.5	37.7
Turkey 6	34.99	16.38	0.13	7.37	0.17	2.62	13.66	75.77
Egypt 2	34.92	16.66	0.13	7.56	0.11	1.93	14.32	75.24
Syria 3	33.98	16.71	0.14	7.68	0.13	2.33	14.22	74.81
Jordan 2	33.88	16.17	0.16	7.76	0.12	2.05	17.01	72.21
Iran 2	33.36	17.26	0.13	6.92	0.04	2.23	18.7	71.43
Portugal 2	32.35	17.09	0.13	6.83	0.03	2.55	16.2	74.03
Egypt 4	32.11	15.78	0.12	7.22	0.1	2.28	14.94	74.59
Remzibey ^C^	31.99	16.96	0.1	6.61	0.05	2.39	20.73	69.46

^C^: Cultivar.

**Table 6:** Genetic parameters for various traits of safflower.

Trait	PV	GV	EV	GCV	GCV.category	PCV	PCV.category	hBS	hBS.category	GAM	GAM.category
Oil (%)	7.65	7.54	0.11	9.31	Low	9.37	Low	98.58	High	19.1	Medium
Protein (%)	0.39	0.38	0	3.83	Low	3.85	Low	99.06	High	7.87	Low
Palmitic	0.16	0.15	0.01	5.31	Low	5.41	Low	96.65	High	10.8	Medium
Palmitoleic	0.01	0.01	0	75.52	High	79.65	High	89.9	High	148	High
Stearic	0.05	0.05	0	9.81	Low	10.08	Medium	94.64	High	19.7	Medium
Oleic	30.3	30.3	0	31.37	High	31.37	High	99.98	High	64.7	High
Linoleic	28.5	28.5	0	7.41	Low	7.41	Low	99.99	High	15.3	Medium

Notes: phenotypic variance (PV), genetic variance (GV), environmental variance (EV), genotypic coefficient of variation (GCV), phenotypic coefficient of variation (PCV), broad-sense heritability (hBS), and genetic advance as a percentage of the mean (GAM).

**Table 7:** Eigenvectors, eigenvalues, and individual and cumulative percentages of variation explained by the first five principal components (PCs) of safflower germplasm.

	F1	F2	F3	F4	F5
Oil (%)	0.368	0.320	0.391	−0.227	0.301
Protein (%)	0.375	0.433	0.181	−0.183	0.297
Miristic	−0.324	−0.215	0.601	−0.182	0.143
Palmitic	−0.421	−0.038	0.549	0.079	−0.097
Palmitoleic	−0.251	−0.342	−0.307	−0.322	0.759
Stearic	−0.157	0.255	0.022	0.827	0.456
Oleic	0.434	−0.484	0.142	0.191	0.042
Linoleic	−0.406	0.496	−0.192	−0.228	−0.068
Eigenvalue	2.644	1.863	1.287	0.983	0.657
Variability (%)	33.054	23.281	16.093	12.287	8.212
Cumulative %	33.054	56.335	72.428	84.716	92.927

## Data Availability

The original contributions presented in the study are included in the article, further inquiries can be directed to the corresponding author.
